# Left-Hemisphere Delay of EEG Potentials Evoked by Standard Letter Stimuli During Rapid Serial Visual Presentation: Indicating Right-Hemisphere Advantage or Left-Hemisphere Load?

**DOI:** 10.3389/fpsyg.2019.00171

**Published:** 2019-02-04

**Authors:** Kamila Śmigasiewicz, Kenneth Wondany, Rolf Verleger

**Affiliations:** ^1^Department of Neurology, University of Lübeck, Lübeck, Germany; ^2^Laboratoire de Neurosciences Cognitives, CNRS, Aix-Marseille Université, Marseille, France; ^3^Institute of Psychology II, University of Lübeck, Lübeck, Germany

**Keywords:** visual evoked potentials, dual-RSVP, RH advantage, LH load, verbal stimuli

## Abstract

During rapid serial visual presentation (RSVP), two streams of letters simultaneously presented in the left and right visual fields (LVF and RVF) evoke visual potentials (VEPs) of EEG a few milliseconds earlier at the right (RH) than the left hemisphere (LH). This small LH VEP lag might be attributed to a RH advantage in initial processing of rapidly changing stimuli or to larger load of the LH by its specialized processing of letters from both visual fields simultaneously. In the present study, the two-stream condition was compared in two experiments to conditions with smaller instantaneous verbal load, namely with stimuli presented either solely or slightly earlier in the LVF or RVF. The RH advantage hypothesis predicts a LH VEP lag very similar to the standard two-stream condition when comparing between LH and RH VEPs contralateral to the single or earlier stream. The LH load hypothesis predicts shorter VEP latencies at the LH in the one-stream and earlier-stream than in the two-stream condition, resulting in an absent LH lag in those conditions. Results tended to be more in line with these latter predictions suggesting that in RSVP the LH might be more involved in partial processing of letters in search for target features. However, since the RH advantage hypothesis could not be reliably rejected these results might indicate a complex interplay between both hemispheres. This interplay would exploit the abilities of either hemisphere during the demanding processing of rapidly presented letters, both the LH advantage in letter processing and the RH advantage in visual perception at initial stages.

## Introduction

The human visual system is faced with multiple dynamically changing sources of stimulation that compete for attention (Desimone and Duncan, 1995). When those sources must be constantly monitored for relevant stimuli to occur, stimuli are at least partially processed in search for target features. The ability to effectively search for targets embedded in a flow of dynamically changing distractors may be studied with the dual-stream rapid serial visual presentation task (*dual-RSVP*). In this task, two streams of stimuli (usually black letters) are presented simultaneously in the left and right visual fields (LVF and RVF), consisting of many standard stimuli and two targets. Rate of stimulus presentation is very fast (130 ms/two stimuli) in order to push the visual system to its limits. The two targets, T1 and T2, may appear randomly in either stream. Thus, both streams must be constantly monitored in search for targets. In this situation, we have observed a right-left asymmetry of visual evoked potentials (VEPs) evoked by the standard stimuli from trial-beginning onwards before any targets were presented: These VEPs, consisting of a sequence of positive–negative peaks (P1 and N1 components) evoked by the sequence of stimuli, appeared slightly earlier over the right hemisphere (RH) than over the left one (LH) (Verleger et al., 2011, 2013; Asanowicz et al., 2017). Both P1 and N1 have generators in extrastriate occipital and temporal (fusiform) cortex (Di Russo et al., 2002; Rossion et al., 2003) and are known to increase by top-down focusing of attention (Heinze et al., 1990; Vogel and Luck, 2000) and to be speeded by brightness (Johannes et al., 1995). Asymmetries of N1 amplitudes between hemispheres have been reported for faces (larger at RH) and words (larger at LH), e.g., by Rossion et al. (2003), but asymmetry of latencies have been much less studied and if so then for unilaterally presented stimuli (Proverbio et al., 2012; Hausmann et al., 2013) whereas stimuli have been presented bilaterally in the present task.

Originally we had assumed that this earlier occurrence of VEPs at the RH than at the LH is related to the well-established large left-right asymmetry in T2 identification in this task. To detail, T1 identification usually is quite effective and equal in both VFs whereas T2 is identified better in the LVF than in the RVF (Holländer et al., 2005; Scalf et al., 2007; Verleger et al., 2009; for review on ensuing studies see Verleger and Śmigasiewicz, 2015; see also Goodbourn and Holcombe, 2015; Holcombe et al., 2017; Ransley et al., 2018, for a similar asymmetry with two simultaneously presented targets). We had speculated that, because the VEP-evoking stimuli precede target presentation in each trial, the RH speeding/LH lagging of VEP latencies might even be causal to, or at least be a forerunner of, the LVF advantage of T2 identification (Verleger et al., 2011), being affected by the same mechanisms.

However, the LH VEP lag has been found to be largely independent of that advantage: First, the measures did not correlate with each other in a relatively large sample of 55 participants (the largest correlation of several VEP measures with asymmetry of T2 identification amounted to *r* = 0.05 only, Asanowicz et al., 2017). Second, shifting attention to the right or left side reduced VEP latencies equally on either side, thus did not interact with the LH VEP lag (Asanowicz et al., 2017; Śmigasiewicz et al., 2017a), in contrast to the marked interaction of attention with the LVF advantage of T2 identification (Śmigasiewicz et al., 2015, 2017a,b). Third, the LVF advantage of target identification was virtually identical in right-handers and left-handers whereas the LH VEP lag was strikingly absent in left-handers (Śmigasiewicz et al., 2017c).

The LH VEP lag was even present when the two stimulus streams were presented above and below fixation (Verleger et al., 2013). This suggests that the RH processes the stimuli faster not only when either hemisphere is occupied by a contralateral stream, but also when both hemispheres must interact to process the two streams of stimuli. Moreover, it was present with different alphanumeric stimulus types. Asanowicz et al. (2017) compared VEPs evoked by familiar (Latin) letters and (Arabic) digits to unfamiliar Tibetan letters 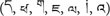. Though smaller for Tibetan letters than for familiar letters and digits, the lag was still present. This pervasiveness of the LH VEP lag, and its absence in left-handers, suggests that it might be an intrinsic feature of hemispheric differences. The question arises what this intrinsic feature might be.

At least two hypotheses may account for this LH lag. First, the RH might be better in constructing percepts at early stages of information processing (Hellige and Webster, 1979; Grabowska and Nowicka, 1996), regardless of other hemispheric specializations at later cognitive stages. Support for this hypothesis comes from studies examining hemispheric differences in processing of visually degraded stimuli. Even if the processing of certain types of stimuli (such as gratings with high spatial frequencies, or as letters or words) is normally left-lateralized their processing under visually degraded conditions is right-lateralized (Polich, 1978; Hellige and Webster, 1979; Hellige, 1980; Sergent, 1983; Johnson and Hellige, 1986; Hellige and Michimata, 1989; Grabowska et al., 1992; for review see: Christman, 1989; Grabowska and Nowicka, 1996). Rapid serial presentation may render the visual stimuli degraded, therefore, according to the RH advantage hypothesis, the RH might have a benefit when processing stimuli during *dual-RSVP*. An alternative hypothesis is based on the evidence that the LH is specialized in processing of verbal information (e.g., Dien, 2009; Dehaene and Cohen, 2011). The RH might perform only some initial, global processing of sensory information of the presented letters and then transmit this information to the LH for more detailed, verbal analysis. Thus, the LH might operate on information from both visual fields. Therefore, the VEPs have longer latencies at the LH than at the RH.

The present study was designed to distinguish between the RH advantage and the LH load hypotheses. To this aim, two experiments were conducted in which we intended to relieve the LH from the putative load imposed by simultaneous presentation. According to the LH load hypothesis, the lag of LH VEPs should disappear under such conditions. According to the RH advantage hypothesis, the lag should still be present.

As a methodological point, it might be suspected that, although rather distant from each other, LH and RH VEP recording sites (PO7 and PO8) are still subject to mutual volume conduction across the scalp which might obscure any time lags between the measured potentials. As in our recent work (Śmigasiewicz et al., 2017a,b) we used Laplacian transformation that redefines potentials as current source densities, by calculating the difference of any potential from the potentials measured at the surrounding recording sites. With this transformation it is unlikely that signals recorded at one hemisphere are contaminated by electrical conduction from the other hemisphere (Kayser and Tenke, 2015; Vidal et al., 2015).

Visual potentials were measured in each trial before any targets were presented. Nevertheless, accuracy of target identification will also be reported, under the questions whether the usual LVF advantage of T2 identification was obtained and whether the experimental manipulations designed to affect VEP latencies evoked by the standard-distractor stream had side effects on target identification.

## Experiment 1

In Experiment 1, aside from the usual two-stream condition, only one stream of stimuli was presented, in either the LVF or the RVF. We assumed that stimuli will be primarily processed by the contralateral hemisphere both in the two-stream and the one-stream condition. VEPs will be compared between contralateral hemispheres in the one-stream conditions, i.e., between the RH from the LVF-stream condition and the LH from the RVF-stream condition. According to the RH advantage hypothesis, VEP latencies will be evoked earlier over the RH than over the LH, like in the two-stream condition. This is because the RH operates independently of the LH and should have better abilities in initial processing of rapidly changing stimulation regardless of simultaneous presence of an ipsilateral stream. To test the LH load hypothesis, the LH will be compared between the two-stream and RVF-stream conditions. According to this hypothesis, the usual additional load of the LH with input from the ipsilateral LVF stream will be absent in the RVF-stream condition, therefore VEPs evoked at the LH will have shorter latencies in the RVF-stream than in the two-stream condition. By this speeding of latencies, the usual LH lag should be absent in the one-stream conditions, when comparing the LH in RVF-stream and the RH in LVF-stream conditions.

### Methods

#### Participants

Sixteen students from the University of Lübeck participated. Two participants’ data had to be rejected from analysis, one because of systematic eye movements toward one stimulus stream (see below, EEG preprocessing) and one because of poor identification of T1 and T2 (both below 2 SDs of the other participants). Although target identification rates were not the objective of the current study, poor identification of targets could result from poor monitoring of stimulus streams and, thus, could have impact on VEPs. This left 14 participants (7 males, mean age = 24.4, SD = 2.9). Before the experiment all participants provided informed written consent and reported normal or corrected-to-normal vision and no history of neurological disorders. After the experiment they received 20 € as compensation. Only right-handed participants were recruited for the study. The average scores in the Edinburgh Handedness Inventory (Oldfield, 1971) amounted to 93 (SD = 13).

#### Stimuli and Apparatus

A computer 16″ CRT screen, driven with 100 Hz, was used for display of stimuli. Participants were seated at about 1.2 m distance from the screen. With this distance 1 cm on the screen spanned 0.5°. Stimuli, displayed on white background, consisted of a central fixation point (a 3 mm × 3 mm small cross) and of capital letters of the Latin alphabet and the digits 1–6 in Helvetica 35 font displayed left and/or right from the fixation point. Letters and digits were 11 mm high and maximally 9 mm wide (their midpoints were 16 mm from fixation). Letters, presented in black, served as standard stimuli. There were two targets embedded in the standard stimuli: the first target (T1) was a blue letter (D, F, G, J, K, L), the second target (T2) was a black digit (1, 2, 3, 4, 5, 6). Presentation^®^ software, version 14.1 (Neurobehavioral Systems Inc.) was used for experimental control.

#### Procedure

The experiment was conducted in a darkened room with the seat in front of the computer screen and a computer keyboard placed on a table. Before the experiment started, participants were instructed to fix their gaze on the fixation point during the entire trial and not to look to the keyboard for entering responses before the response prompt appeared on the screen. Each trial started with the fixation cross that lasted until the end of the stimulus stream. 800 ms after fixation point onset, the first frame of distractor stimuli was presented.

**FIGURE 1:**
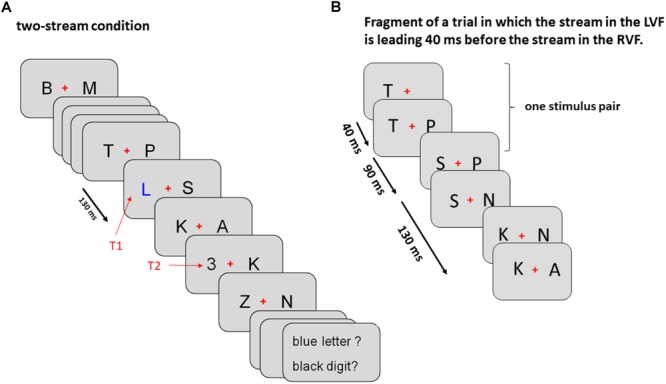
Sequence of events in Experiment 1 **(A)** and Experiment 2 **(B)**. **(A)** The sequence of events in Experiment 1 is exemplified for a left T1 – left T2 sequence in the two-stream condition. In the LVF condition stimuli were displayed only on the left from the fixation cross and in the RVF condition only on the right from the fixation cross. Onsets of consecutive stimulus frames are 130 ms apart, as indicated by the black arrow on the left. **(B)** Initial part of both streams of standard stimuli in Experiment 2 encompassing first three stimulus pairs (six frames; see section “Methods” for details). In this example the left stream was leading 40 ms before the right stream.

As illustrated by Figure 1A, trials were divided in three tasks presented in separate blocks. (1) In the two-stream task, stimuli were displayed both left and right from fixation, (2) in the LVF-stream task stimuli were solely presented on the left side and (3) in the RVF-stream task stimuli were solely presented on the right side. All stimulus frames were appearing for 130 ms and were immediately followed by the next frame. T1 was presented randomly as 6th, 8th, or 10th frame and T2 randomly as 1st or 4th frame after T1 (lags 1 or 4, 130, or 520 ms after T1 onset). T2 was followed by five frames of stimuli. Thus, overall, between 12 and 19 frames of stimuli were displayed during one trial. In the two-stream task both targets could appear in the left or right stream and were accompanied by a standard letter in the opposite stream. In the one-stream tasks both targets could appear only in one and the same stream.

Targets were randomly selected from the target sets, standards were randomly selected with replacement from the standard letter set (with a restriction against immediate repetition and against equal characters simultaneously in the left and right stream). At the end of each trial, the fixation cross terminated, and participants were asked to enter their responses on a standard keyboard, first the T1 letter on the middle row and then the T2 digit on the number pad. They were asked to make their best guess in case the answer was not known. The next trial started immediately after the T2 response.

Eye position was measured by means of a remote infrared eye-tracker (600 series binocular, Eyegaze LC Technologies, Fairfax, VA, United States) and online fed back by software (Interactive Minds, Dresden, Germany) which communicated with the Presentation program. At the beginning of each trial, fixation was checked by the program. In case of a deviation of more than 6 mm from vertical midline, a red exclamation mark was presented at midline, inducing shifts of gaze back to fixation.

Each of the three blocks (two, LVF, RVF streams) consisted of 240 trials, in random order of occurrence evenly distributed across the 24 combinations of trial construction in the two-stream condition (three T1 time-points × two lags between T1 and T2 × two T1 sides × two T2 sides) and the six combinations in each one-stream condition (where T1 side and T2 side were fixed). Order of the three conditions across the 14 participants was almost balanced, by having each of the six possible orders of the three task blocks in at least two participants. In both experiments, before the task proper, about 10 trials were presented in slow motion (500 ms) and 10 with original presentation rate (130 ms) for practice. There were also two breaks which were introduced after every 240 trials.

#### EEG Recording and Pre-processing

Ag/AgCl electrodes (Easycap ^1^) were used to record EEG. They were placed at 60 scalp sites which were 8 midline positions from AFz to Oz and 26 pairs of symmetric left and right sites, and at the nose-tip. On-line reference was placed at Fz, and after recording, data were re-referenced to the nose-tip. Fpz was connected to ground. Four electrodes placed around the eyes were used to control for eye movements: two electrodes above and below the right eye for vertical electro-oculogram (vEOG) and two at the outer canthi for horizontal EOG (hEOG). Data were amplified from DC to 250 Hz by a BrainAmp MR plus and stored at 500 Hz per channel. Data preprocessing and analysis was done with Brain-Vision Analyzer software (version 2.02).

For analysis of VEPs the low-pass filter was set at 20 Hz and segments were defined as starting 100 ms before onset of the first distractor pair and extending until 900 ms afterwards. All trials were included irrespective of correctness of target identification. Editing for artifacts consisted of rejecting trials with zero lines, with overall minimum-maximum voltage differences ≥ 200 μV and with voltage steps between adjacent data points ≥ 50 μV. Additionally, data were high-pass filtered at 3 Hz in order to remove slow drifts from the signal. Finally, data were referred to the first 100 ms before the first standard letter pair as baseline. Horizontal eye movements indicate unequal monitoring of the LVF and the RVF. In order to reject participants with relevant proportion of such eye movements, hEOG was formed as averages of differences contralateral-ipsilateral to T1 and subsequently inspected for T1-induced saccades, defined as deviations from baseline by 10 μV within 700 ms after T1 onset, indicating eye movements ≥ 0.7° toward T1. One participant was rejected based on this criterion (see above).

Subsequently, averaged data were spatially filtered using the current source density (CSD) approach. CSD accentuates local effects while filtering out distant effects due to volume conduction (Burle et al., 2015; Kayser and Tenke, 2015). All averaged ERP waveforms at each electrode were transformed into reference-free CSD estimates (μV/m^2^) using the surface Laplacian algorithm with interpolation by spherical splines (Perrin et al., 1989, 1990) implemented in Brain Vision Analyzer software with the following computation parameters: order of spline 4; maximal degree of Legendre polynomials 10; approximation parameter lambda 1.0e-005.

#### Data Analysis

The streams of standard stimuli evoked a series of P1 and N1 potentials from the beginning of the trial, appearing in the rhythm of stimulus presentation, every 130 ms. These VEPs and their CSD transforms were largest at PO8 and PO7 and therefore these recording sites were used to measure the lags between CSD-transformed VEPs evoked at the LH and at the RH. Each participant’s waveforms at PO8 and PO7 were shifted against each other in 2 ms steps within ±66 ms (i.e., ±half the onset interval between stimuli) and the shift that rendered the largest cross-correlation was selected (Okon-Singer et al., 2011). Analysis included 800 ms of the wave duration, encompassing five P1 and N1 peaks. (The sixth N1 peak could already contain T1). First, VEP lags were tested against zero by *t*-tests between hemispheres in the two-stream condition and between contralateral hemispheres (receiving direct input from the presented streams) in LVF-stream and RVF-stream conditions. Second, the same waveforms were arranged into different pairs, for comparing the two-stream condition with the contralateral hemisphere in the one-stream condition and testing the lag against zero, separately for the RH and the LH. Third, VEP lags were tested against zero within the two one-stream tasks separately, between the contralateral (receiving direct input) and ipsilateral (not receiving direct input) hemispheres. Thus, within each of these three sets of four waveforms, two lags were computed and either lag was tested against zero. Additionally, these two lags were also tested against each other. Thus, within each of the three sets, three *t*-tests were performed.

To quantify behavior, percentages of trials with correct responses were computed in each condition (pooled across the three T1 time-points) for T1 accuracy and for T2| T1 accuracy. Accuracy rates of T1 and T2 identification were each entered to analyses of variance (ANOVAs). To compare dual and one-stream tasks, identification rates of the dual-stream task were analyzed only when T1 and T2 were in the same stream because this was the only way targets could be presented in the one-stream tasks. Results from the different-stream condition of the dual-stream task will be reported in tables and figures, too, but will not be further analyzed. ANOVAs, therefore, had the factors Number of Streams (one vs. two), Lag between T1 and T2 (1, 4), and Target Side (left, right; target being T1 or T2 depending on the analysis). Only the main effect and interactions of Stream Number will be reported in detail because this is the new manipulation relative to our previous, above-quoted papers.

### Results

#### Visual Potentials (VEPs)

**FIGURE 2:**
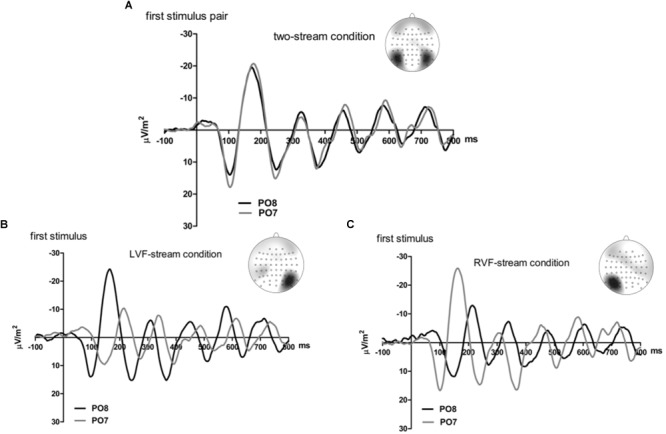
Comparison of visual evoked potentials between right and left scalp sites in Experiment 1. Displayed are grand means of CSD transforms of visual potentials evoked by the streams of left and/or right standard stimuli during the first 800 ms of the trial in the two-streams condition **(A)**, in the LVF-stream condition **(B)**, and in the RVF-stream condition **(C)**. In each panel, CSDs are shown from the right scalp site PO8 (black lines) and the left scalp site PO7 (gray lines). The topographical maps show the scalp topography at the time-point of the peak of the first N1 component.

**FIGURE 3:**
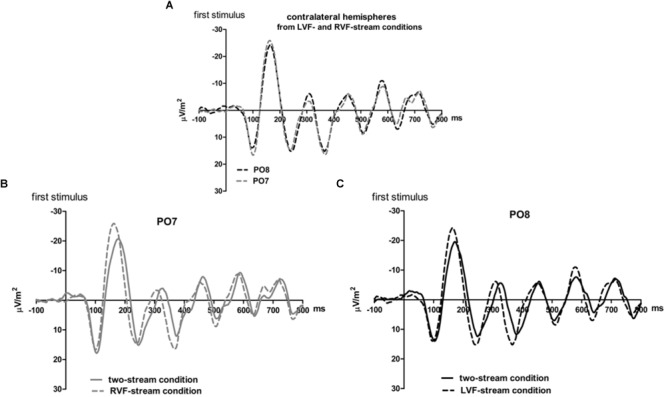
Comparison of visual evoked potentials between tasks in Experiment 1. Displayed are grand means of CSD transforms of visual potentials evoked during the first 800 ms of the trial at sites contralateral to the unilateral streams, and compared with each other **(A)** or with potentials evoked at the same site in the bilateral task **(B,C)**. Thus, panel A displays waveforms from the contralateral hemispheres in the two unilateral conditions, RVF stream (PO7, i.e., LH) and LVF stream (PO8, i.e., RH). **(B,C)** Displays waveforms from the LH and the RH, respectively, comparing unilateral with bilateral tasks. Waveforms from PO8 are in black, from PO7 in gray, solid lines denote the two-stream condition and dashed lines the condition with one stream.

Figure 2, 3 present grand-average CSD waveforms of the first 800 ms from the beginning of the letter stream, recorded at PO8 (RH) and PO7 (LH). Statistics about the lags are compiled in the left part of Table 1. In the two-stream condition (Figure 2A), VEPs were evoked earlier at PO8 than PO7 by 7 ms on average (*t*_13_ = -2.2, *p* = 0.04). This LH lag was reduced to non-significance in the one-stream conditions when comparing PO8, contralateral to the left stream, with PO7, contralateral to the right stream (Figure 3A), where VEPs occurred only 2 ms earlier in the RH (PO8) than in the LH (PO7) (*t*_13_ = -1.2, *p* = 0.26). The difference between the two lag differences did not reach the *p* = 0.05 level, though (7 ms vs. 2 ms; *t*_13_ = -1.8, *p* = 0.09).

**Table 1:** Lags (positive) or leads (negative) of one waveform compared to another one as indicated in each row.

Experiment 1				Experiment 2			
	Mean (ms)	*SD* (ms)	*t, p*		Mean (ms)	*SD* (ms)	*t, p*
2 streams, PO8–PO7	–6.9	11.4	**–2.2 0.04**	Simult. streams PO8–PO7	–8.0	14.5	–**2.3 0.04**
1 stream, LVF or RVF PO8(LVF)–PO7(RVF)	–1.9	5.8	–1.2 0.26	One stream leading PO8(LVF)–PO7(RVF)	–3.1	9.5	–1.3 0.20
Difference	–5.0	10.4	–1.8 0.09	Difference	–4.9	8.2	–**2.5 0.02**
PO7(RVF) 2–1 streams	6.9	9.3	**2.7 0.02**	PO7(RVF) Simult.–leading RVF	2.7	4.8	**2.3 0.04**
PO8(LVF) 2–1 streams	3.3	7.5	1.6 0.13	PO8(LVF) Simult.–leading LVF	1.8	5.4	1.4 0.20
Difference	3.6	10.0	1.3 0.21	Difference	0.9	7.7	0.5 0.62
1 stream, LVF PO8–PO7	38.0	18.3	**7.8 <0.001**	LVF leading PO8–PO7	39.9	10.8	**15.2 <0.001**
1 stream, RVF PO7–PO8	37.9	16.2	**8.7 <0.001**	RVF leading PO7–PO8	30.6	18.1	**6.9 <0.001**
Difference	0.1	24.9	0.0 0.98	Difference	9.3	22.3	1.6 0.10

*Equivalent comparisons from Experiments 1 and 2 are in the same rows. Values are presented as means and SDs across participants and, in the rightmost columns of the left and right halves, as t-values and their probabilities. These values are printed in bold when *p* < 0.05.*

Correspondingly, when comparing two-stream with one-stream conditions separately for either hemisphere (Figure 3B,C) RVF-VEPs at PO7 (LH) were evoked later in the two-stream than in the one-stream condition (by an average difference of 7 ms; *t*_13_ = 2.7, *p* = 0.02) whereas the same effect was not significant for LVF-VEPs evoked at PO8 (RH) (a difference of 3 ms on average: *t*_13_ = 1.6, *p* = 0.13). This difference between PO7 and PO8 in the amount of the one vs. two-stream difference was not significant, though (7 ms vs. 3 ms; *t*_13_ = 1.3, *p* = 0.20).

Besides, VEPs recorded from ipsilateral recording sites in the one-stream conditions drastically lagged behind their contralaterally recorded VEPs by mean lags of 38 ms, both when the stimulus stream was presented in the LVF only (Figure 2B), *t*_13_ = 7.8, *p* < 0.001, and when the stream was presented in the RVF only (Figure 2C), *t*_13_ = -8.7, *p* < 0.001, without significant difference (*t*_13_ = 0.0, n.s.).

#### Identification Rates

**FIGURE 4:**
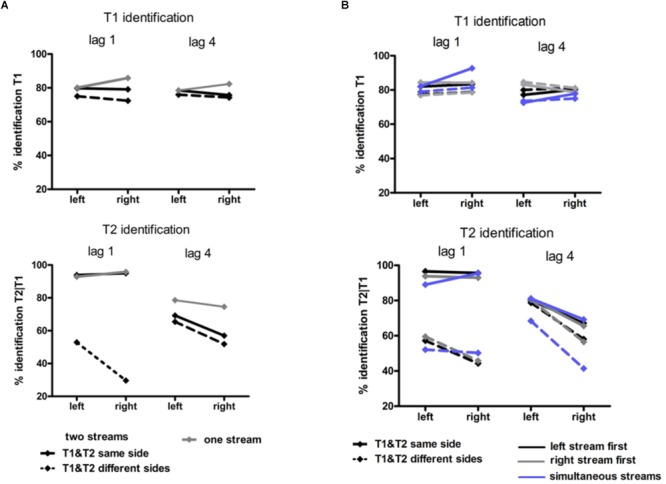
Behavioral results in Experiment 1 **(A)** and Experiment 2 **(B)**. Identification rates for T1 (upper graphs) and for T2 (lower graphs). T1 accuracy was calculated as the percentage of correct T1 relative to all trials. T2 accuracy was calculated as the percentage of correct T2 in those trials where T1 was correctly identified. In Experiment 1 **(A)** black lines represent the two-stream condition in which T1 and T2 were presented in the same stream (solid lines) and in different streams (dashed lines). Gray lines represent the one-stream condition in which the stream was presented in the RVF or in the LVF. In Experiment 2 solid lines represent conditions in which T1 and T2 were presented in the same stream and dashed lines conditions with T1 and T2 presented in different streams. Blue lines represent conditions with two streams simultaneously presented left and right, black lines conditions with the stream on the left being presented earlier than the stream on the right and gray lines conditions with the stream on the right being presented earlier than the stream on the left.

Identification rates are compiled in Table 2 and presented in Figure 4A. As mentioned in the method section, only effects of Stream Number will be reported in detail, and identification rates of dual-stream presentation were analyzed from same-stream conditions only, for comparison with the one-stream tasks where targets could be presented on the same side only.

**Table 2:** Percentages of correct identification of T1 (relative to all trials) and of T2 (relative all T1-correct trials) in Experiment 1.

Lag		1				4			
T1T2 stream		Same		Different		Same		Different	
Target side		LVF	RVF	LVF	RVF	LVF	RVF	LVF	RVF
Two-streams	T1	80 (15)	79 (20)	75 (16)	72 (23)	79 (15)	76 (20)	76 (18)	74 (20)
	T2	94 (11)	95 (10)	53 (28)	30 (21)	69 (22)	57 (25)	66 (31)	52 (25)
LVF-stream	T1	80 (22)				79 (19)		
	T2	93 (11)				78 (22)		
RVF-stream	T1		86 (17)				82 (18)	
	T2		96 (7)				75 (22)	

*Values are presented as means across participants (SD). Values shaded in gray (different-side targets in the two-stream condition) did not enter the ANOVA that tested for the Stream factor*.

##### T1 identification

It might be inferred from Figure 4A (upper graph) that there was an interaction of Stream Number and Target Side. This effect was not significant, though (*F*_1,13_ = 3.4, *p* = 0.09) nor was there any other effect of Stream Number.

##### T2 identification

As may be seen in Figure 4A (lower graph), as usual a LVF advantage was obtained except for same-side lag 1 (likewise as usual) where identification rates were almost perfect. (ANOVA effects not detailed, for brevity). Stream Number had an effect with lag 4 where identification rates were worse with two streams than with one stream, Lag × Stream Number *F*_1,13_ = 7.7, *p* = 0.02.

### Discussion

As predicted by the LH load hypothesis, VEPs measured at the LH became faster when there was no ipsilateral stream, such that the difference between hemispheres was not significant any more in the one-stream conditions. Yet the lag differences were quite variable across participants (cf. SDs in Table 1) such that differences between lag effects were not significant. Therefore, these results certainly need replication.

Moreover, the two-stream and one-stream conditions might principally differ in the way both hemispheres operate during the task (independent or concurrent processing in the two-stream condition and rather cooperative processing in the one-stream condition) and the way attention is distributed between left and right streams (fully unilaterally in one-stream conditions, diffusely bilaterally or alternating between sides in two-stream conditions).

In view of those open issues we conceived of a second experiment that should replicate the essential features of Experiment 1 but should use bilateral stimulation in all conditions.

## Experiment 2

Experiment 2 aimed at replicating the one-stream results with two-stream presentation. Aside from the condition in which both streams were presented simultaneously, stimuli were presented slightly earlier (with lags varying between 20 and 60 ms) either in the LVF than in the RVF (the leading-LVF condition) or vice versa (the leading-RVF condition). (This lagged presentation mode was inspired by a study by Clement and Matthews, 2016). The predictions were similar to Experiment 1. With simultaneous presentation of both streams, the usual lag of LH VEP latencies was expected. According to the RH advantage hypothesis, this lag should still be present when comparing contralateral hemispheres between leading-LVF and leading-RVF conditions. According to the LH load hypothesis, the LH will handle the load of the input from both visual fields better when stimuli are presented one after the other rather than simultaneously. Accordingly, the VEP latencies evoked at the LH might be shorter with RVF leading than with simultaneous streams, making the LH VEP lag disappear.

### Methods

Only the differences from Experiment 1 will be described.

#### Participants

Eighteen students from the University of Lübeck participated. One participant’s data had to be rejected from analysis because of poor identification of T1 and T2 (both below 2 SDs of the other participants). This left 17 participants (9 males, mean age = 22.4, *SD* = 2.5) with average scores in the Edinburgh Handedness Inventory (Oldfield, 1971) of 90 (*SD* = 16).

#### Procedure

There were three conditions presented randomly within the same block of trials. (1) The simultaneous-stream condition was the standard condition. (2) In the leading-LVF condition, stimuli were presented earlier in the LVF than in the RVF, by constant lags of 20, 30, 40, 50, or 60 ms, varying between trials. (3) Correspondingly, in the leading-RVF condition, stimuli were presented earlier in the RVF than in the LVF by constant lags of 20, 30, 40, 50, or 60 ms. Technically, these lags between streams were achieved by presenting twice as many frames as in the simultaneous-stream condition. As illustrated in Figure 1B for the leading-LVF condition, in the first frame a letter was displayed only in the LVF. After 20, 30, 40, 50, or 60 ms this frame was replaced by a new frame in which still the same letter as in the previous frame was displayed on the left and additionally a new letter was displayed on the right. These two frames were presented for 130 ms altogether. Thus, when the first frame was presented for 20 ms, the second one was presented for 110 ms, and when the first one was presented for 60 ms, the second one was presented for 70 ms. These alternating presentation times were constant within a trial. The third frame contained a new letter on the left and the same letter as in the previous frame on the right and lasted for the same time as the first frame, and the fourth frame contained a new letter on the right and the same letter as in the previous frame on the left and lasted for the same time as the second frame. This scenario with double frames repeated as many times as there were stimulus-pairs in the trial. In consequence, within a given trial the letter on the left was always presented slightly earlier than the letter on the right. In all three conditions, both targets could randomly appear in either stream. Like in Experiment 1, T1 was presented within the 6th, 8th, or 10th frame-pair, T2 within the 1st or 4th frame-pair after T1, and the T2 frame was followed by five frame-pairs.

The Stream variable had 11 levels randomly chosen in each trial: (1) both streams simultaneously, (2–6) stream in the LVF leading by 20, 30, 40, 50, or 60 ms; and (7–11) stream in the RVF leading by 20, 30, 40, 50, or 60 ms. The simultaneous condition was replicated 70 times, and each of the other ten levels was replicated 65 times, yielding 720 trials. Independently of stream level, the 24 combinations of T1 and T2 occurrence (as specified with Experiment 1) were again evenly distributed across the 720 trials in random order.

#### Data Analysis

Analysis of VEP lags included 860 ms of the wave duration (i.e., 800 ms plus the maximum lag between VFs of 60 ms). In statistical testing of the lags, the two-stream, single-LVF stream, and single-RVF stream conditions of Experiment 1 now corresponded to simultaneous, leading-LVF, and leading-RVF stream conditions.

In ANOVAs on accuracy rates of T1 and T2 identification, the full data set could be entered to ANOVA (different from Experiment 1) where it was analyzed with the factors Stream Timing (leading LVF, simultaneous, leading RVF), Lag (1, 4), Target Side (left, right; target being T1 or T2 depending on the analysis) and Other-Target Side (same, different). When T1 was analyzed, Other-Target Side referred to the side of presentation of T2 and conversely, when T2 was analyzed, Other-Target Side referred to the side of presentation of T1. Only the main effect and interactions of Stream Timing will be reported in detail. Having three levels, these effects were corrected by the Greenhouse–Geisser method.

### Results

**FIGURE 5:**
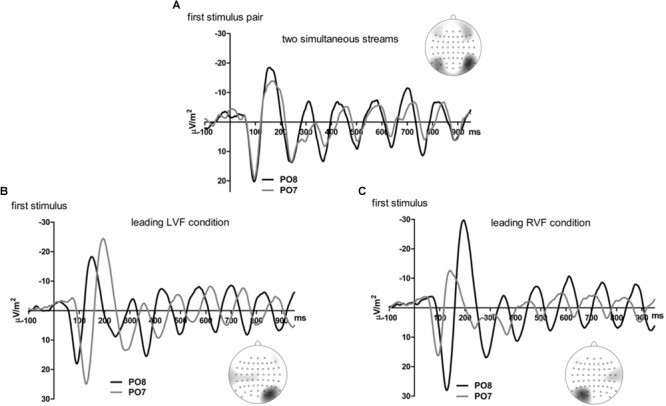
Comparison of visual evoked potentials between right and left scalp sites in Experiment 2. Displayed are grand means of CSD transforms of visual potentials evoked by the stream of left and right standard stimuli during the first 950 ms of the trial in the simultaneous-stream condition **(A)**, in the leading-LVF condition **(B)** and in the leading-RVF condition **(C)**. In each panel, CSDs are shown from the right scalp site PO8 (black lines) and the left scalp site PO7 (gray lines). The topographical maps show the scalp topography at the time-point of the peak of the first N1 component.

**FIGURE 6:**
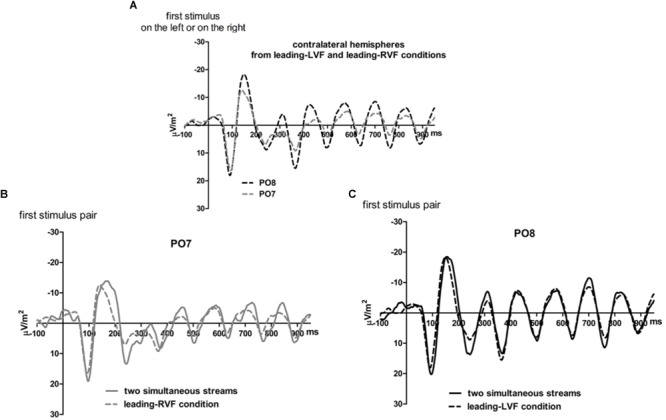
Comparison of visual evoked potentials between tasks in Experiment 2. Displayed are grand means of CSD transforms of visual potentials evoked during the first 950 ms of the trial at sites that lead in the leading-LVF and leading-RVF conditions, and compared with each other **(A)** or with potentials evoked at the same site when streams were simultaneous **(B,C)**. Thus, **(A)** displays waveforms from the contralateral hemispheres in the leading-RVF (PO7, i.e., LH) and the leading-LVF (PO8, i.e., RH) conditions. **(B,C)** Displays waveforms from the LH and RH, respectively, comparing unilateral with bilateral tasks. Waveforms from PO8 are in black, from PO7 in gray, solid lines denote the simultaneous-stream condition and dashed lines the conditions with one leading stream.

Figure 5, 6 present grand-average CSD waveforms of the first 950 ms from the beginning of the letter stream, recorded at PO8 and PO7. Statistics about the lags are compiled in the right half of Table 1. In the simultaneous-stream condition (Figure 5A), VEPs were evoked earlier at PO8 than PO7 by 8 ms on average (*t*_16_ = -2.3, *p* = 0.04). This LH lag was reduced to non-significance when comparing PO8 with PO7 as contralateral sites to the leading stream, with VEPs occurring 3 ms earlier in the RH (PO8) than in the LH (PO7) (Figure 6A; *t*_16_ = -1.3, *p* = 0.20). The difference between the two lag differences was indeed significant; 8 ms vs. 3 ms: *t*_16_ = -2.5, *p* = 0.02.

Correspondingly, when comparing simultaneous with leading-stream conditions for the site contralateral to the leading stream (Figure 6B,C), RVF-VEPs at PO7 (LH) were evoked later in the simultaneous-stream condition than in the leading-RVF condition by 3 ms (*t*_16_ = 2.3, *p* = 0.04) whereas the corresponding effect (simultaneous condition versus leading LVF) was not significant for PO8 (RH) (1.8 ms, *t*_16_ = 1.4, *p* = 0.20). This difference between PO7 and PO8 in the amount of the simultaneous vs. one-VF-leading difference was not significant, though (3 ms vs. 1.8 ms; *t*_16_ = 0.5, *p* = 0.62).

Besides, in the one-stream-leading conditions, VEPs recorded from ipsilateral recording sites drastically lagged behind their contralaterally recorded VEPs, by a mean lag of 40 ms when the LVF stream was leading (Figure 5B) (*t*_16_ = 15.2, *p* < 0.001) and by a mean lag of 31 ms in the leading-RVF condition (Figure 5C) (*t*_16_ = 6.9, *p* < 0.001), without significant difference (*t*_16_ = 1.6, *p* = 0.10).

#### Identification Rates

Identification rates are compiled in Table 3 and presented in Figure 4B. As mentioned in the method section, only effects of Stream Timing will be reported in detail.

**Table 3:** Percentages of correct identification of T1 (relative to all trials) and of T2 (relative all T1-correct trials) in Experiment 2.

Lag		1				4			
T1T2 stream		Same		Different		Same		Different	
Target side		LVF	RVF	LVF	RVF	LVF	RVF	LVF	RVF
Simultaneous streams	T1	82 (22)	93 (14)	79 (18)	81 (23)	73 (25)	78 (16)	74 (30)	75 (29)
	T2	89 (21)	95 (8)	52 (36)	50 (30)	81 (27)	63 (29)	68 (37)	41 (40)
Leading-LVF	T1	82 (12)	83 (13)	77 (14)	79 (15)	77 (17)	80 (13)	80 (15)	81 (16)
	T2	97 (6)	96 (8)	57 (22)	44 (25)	80 (14)	67 (17)	79 (17)	58 (21)
Leading-RVF	T1	84 (11)	84 (14)	77 (18)	79 (16)	83 (14)	79 (16)	85 (11)	81 (14)
	T2	94 (7)	93 (7)	59 (24)	46 (22)	81 (14)	66 (21)	80 (13)	57 (23)

*Values are presented as means across participants (SD)*.

##### T1 identification

There was no significant effect of Stream Timing (all *F*_2,32_ ≤ 3.2, *p* ≥ 0.09). Inspection of Figure 4B (upper graph) might suggest otherwise, yet the simultaneous condition (where some mean values seemed to deviate from the left- or right-leading conditions) consisted of 70 trials only, such that there were only few trials for each of the eight conditions within the simultaneous condition, making differences between LVF and RVF in this condition noisy.

##### T2 identification

Figure 4B (lower graph) shows the usual LVF advantage but suggests that there was no such advantage with simultaneous streams and targets on the same side. Yet, in fact the LVF advantage was not significantly modified by Stream Timing, all interactions of Stream Timing × Target Side *F*_2,32_ ≤ 2.5, *p* ≥ 0.10. With regard to other effects of Stream Timing, Figure 4B (lower graph) suggests that in the condition in which T2 was presented at lag 4 on the other side than T1, T2 was identified least correctly with simultaneous streams. However, the interaction between Stream, Lag and Other-Target Side did not reach significance (*F*_1,16_ = 3.0, *p* = 0.08). The deviating mean values of simultaneous streams and/or their lacking statistical significance are probably a consequence of the mentioned variability due to the low number of trials in the simultaneous-stream condition.

### Discussion

Results of Experiment 1 were very well replicated. (Cf. the highly similar pattern of right and left sides of Table 1). This replication was achieved even though temporal shifting of the streams is a softer manipulation than omitting one stream altogether and even though temporal shifts occurred unpredictably across trials whereas the one-stream conditions of Experiment 1 were presented in separate blocks. Thus, results of the two experiments will be together discussed in the following section.

## General Discussion

### Predictions

The present experiments were designed to elucidate the mechanism underlying the difference of VEP latencies between the right and left hemispheres (LH VEP lag) evoked by rapid streams of letters during *dual-RSVP*. To this aim, in addition to the usual two-stream condition, conditions including only one stream presented in the left or right visual field were administered in Experiment 1. To avoid possible confounds of the one-stream conditions resulting from unequal perceptual load and unilaterally directed attention, in Experiment 2 stimuli were presented slightly earlier in one than in the other visual field. It was assumed that lack or latecomer presentation of the other stream will facilitate processing of the only present or earlier present stream. This assumption was relevant for the LH load hypothesis which stated that the reason for the LH VEP lag is that the LH, being specialized for processing verbal input, processes character-symbol input from both visual fields. Thus, it was expected that VEP latencies at the LH will be shorter when a stream will be presented only in the RVF than in the condition with both streams. In contrast, according to the RH advantage hypothesis, the LH VEP lag will be present no matter whether one or two streams will be presented. This is because when either hemisphere is involved in processing only its contralateral visual input, the RH will always have an advantage over the LH in processing degraded stimuli at early stages. Presence or absence of some stream in the ipsilateral field should not have an impact on the way the RH processes its contralateral stimuli.

### Outcomes

In the two-stream condition of Experiment 1 and the simultaneous-stream condition of Experiment 2, VEPs at the LH lagged behind the RH, by 7 and 8 ms, respectively. This LH VEP lag replicated our previous findings (Verleger et al., 2011; Asanowicz et al., 2017). Results of the experimental conditions tended to be in favor of the LH load hypothesis. In Experiment 1, VEPs recorded at the LH were evoked earlier (by 7 ms on average) when only the contralateral stream was presented than with both streams. This suggests that in the one-stream condition the LH was relieved from processing the visual input from both visual fields and therefore could process the information from the contralateral field more effectively. Similar results were obtained in Experiment 2: VEPs at the LH were evoked 3 ms earlier in leading-RVF than in simultaneous-stream trials. This effect appeared smaller than in Experiment 1, possibly because the LH was still involved in processing information from both visual fields, suggesting that the processing load, though indeed being smaller when stimuli were presented alternatingly in both streams than simultaneously, was still larger than when only one (contralateral) stream was presented.

The analogical effect of speeded latencies for the RH, although present (3 and 1.8 ms in Experiments 1 and 2), did not reach significance. In consequence of this lesser speeding, the LH lag was absent for the contralateral hemispheres in the one-stream and one-leading stream conditions, which disconfirms the prediction based on the RH advantage hypothesis according to which the RH advantage in processing degraded stimuli should occur throughout, because there is no reason why this ability should become worse when the task gets simpler. However, it may be argued in favor of this hypothesis that the RH advantage is actually a LH disadvantage. Accordingly, in the one-stream condition of Experiment 1 the LH may compensate for its disadvantage of perceiving degraded stimuli by focusing attention on its contralateral RVF whereas the RH does not need much attention for identifying the stimuli in its contralateral LVF. Therefore, since directing attention to one side speeds contralateral VEP latencies in this task (Asanowicz et al., 2017; Śmigasiewicz et al., 2017a) the LH VEPs will become more speeded than the RH VEPs. However, this hypothesis cannot account for the speeding of LH VEPs in the critical right-leading condition of Experiment 2. This is so because there is no reason why participants should focus attention on the right stream in this experiment: Either target appeared unpredictably with equal probability in one of the two streams in Experiment 2 irrespective of whether the right stream led the left one or vice versa. Indeed, identification rates and the LVF advantage in T2 identification were not affected by whether the left or right stream was the leading one, suggesting that the variation of lags between left and right stimulus onsets did not affect the directing of attention. Nevertheless, this variation of stimulus lags affected VEP lags more at the LH than the RH, in good accordance with the LH load hypothesis. Taken together, the current results speak more for the LH load hypothesis than for the RH advantage hypothesis, although alternative interpretations cannot be firmly refuted, as will be discussed below.

A plausible assumption is that this load of the LH is related to the LH’s verbal competences. The LH is more engaged than the RH in processing of verbal stimuli, such as letters or words (Dien, 2009; Dehaene and Cohen, 2011). The question arises why then, in Asanowicz et al. (2017) study, a LH VEP lag was present, albeit reduced, even when the streams consisted of Tibetan letters. If, as was assumed in that study, Tibetan letters are unfamiliar, non-verbal stimuli, there should be no LH lag at all according to LH load hypothesis. Consequently, in that study, the LH lag was interpreted as showing a RH advantage in processing of degraded stimuli at early stages. However, it may be argued that the RH advantage hypothesis would not predict a diminished but rather an increased LH lag with unfamiliar stimuli due to enhanced difficulty in their processing. Moreover, it may be asked whether Tibetan letters would really be processed as non-verbal stimuli. Without applying brain imaging methods, it is difficult to verify where in the brain, and thus how, stimuli are processed (cf. Szwed et al., 2014). Even with “non-verbal” stimuli, participants might use some strategies to treat them as “verbal” and thereby facilitate their processing and memorization. In this line, though being less verbal stimuli than Latin letters or Arabic digits Tibetan letters might still possess verbal features which may be the reason why the LH lag was not absent but reduced in VEPs evoked by such stimuli, as would be predicted from the LH load hypothesis.

However, there are limitations in interpreting the current results uniquely in favor of the LH load hypothesis. True, in favor of this hypothesis, the speeding of latencies in the one-stream or earlier-stream conditions compared to the two-stream condition was significant for the LH only. However, although being insignificant at the RH, latency speeding did not significantly differ between RH and LH either. Thus, one might argue that some general factors distinguishing the one-stream or earlier-stream conditions from the standard two-stream condition, such as overall diminished perceptual load, lack of competition from the other hemisphere, or even the cooperation received from the other hemisphere might have participated in speeding VEP latencies to similar extents in both hemispheres. In spite of this ambiguity, we tend to interpret the results in favor of the LH load hypothesis since the speeding was significant for the LH only, replicable in two experiments.

### Relationship Between LH Lag and Callosal Transfer

Alternatively to the LH load and RH advantage hypotheses, the LH VEP lag might be attributed to unequal speed of information transfer between hemispheres. Indeed, a large part of studies on interhemispheric transfer time (IHTT), measured by reaction times or VEP latencies, indicates that the transmission of verbal (Brown and Jeeves, 1993; Brown et al., 1994; Larson and Brown, 1997) and non-verbal information (Rugg et al., 1984; Marzi et al., 1991; Larson and Brown, 1997; Patston et al., 2007; Siman-Tov et al., 2007; Okon-Singer et al., 2011) is asymmetric. However, this asymmetry consists of faster transmission from the RH to the LH than vice versa. Thus, if due to this asymmetry in IHTT, VEPs should be evoked earlier in the LH rather than in the RH, opposite to the actual LH lag.

With two-stream stimulation, as used in *dual RSVP*, callosal transfer is hard to measure, though. This is so because VEPs in either hemisphere are primarily due to direct stimulation from the contralateral VF and may be only secondarily affected by callosal transfer from the other hemisphere. Therefore, the purest measure of IHTT was obtained in our experiments by the one-stream tasks in Experiment 1. Here, the difference between contralateral waves (evoked at the hemispheres that could “see” the letters) and ipsilateral waves (recorded at the hemisphere that could not “see” the letters which, therefore, had to be transferred through the corpus callosum or anterior commissure from the other hemisphere) did not differ between LVF- and RVF-stream conditions, amounting to 38 ms both from RH to LH (LVF stream) and from LH to RH (RVF stream) (see Table 1). Therefore, this result does not point to unequal transfer speed of information between hemispheres as a cause of the LH VEP lag.

This symmetric IHTT is in contrast to several other studies mentioned above. As concluded by Nowicka and Tacikowski (2011), IHTT depends on many factors such as group tested, stimulus type, or the required cognitive operation. Actually, in studies on IHTT the task usually required detecting or identifying the stimuli. This is not the case in the current experiment, where participants only had to monitor the stream of standard letters in search for some partial matching with a target template. This may involve cognitive operations different from deliberate stimulus detection or identification and might have led to a distinct pattern of the asymmetry in IHTT. Unfortunately, Experiment 2 did not help in resolving this conflict. On the contrary, if it is assumed that callosal transfer was measured in the one-VF-leading conditions of Experiment 2 as well as in the one-VF conditions of Experiment 1, then the results obtained in Experiment 2, with 40 ms difference from RH to LH but only 31 ms difference between LH and RH, although not statistically significant (*p* = 0.10 only, cf. Table 1) were numerically just opposite to the usual faster IHTT from RH to LH.

## Conclusion

Results of the two experiments conducted in the current study suggest that the LH lag of VEP latencies, as evoked by the stream of standard letters in *dual-RSVP*, is related to load of the LH with processing of alphanumeric input from both visual fields. VEPs were evoked earlier at the LH when it was either entirely released from processing the additional stream presented in the ipsilateral field or when the stimuli in the ipsilateral stream were presented slightly later than in the contralateral stream allowing for alternating processing of stimuli from both visual fields. By this speeding of LH VEPs, the LH lag was abolished.

## Ethics Statement

This study was carried out in accordance with the recommendations of the Ethical Committee of the University of Lübeck with written informed consent from all subjects. All subjects gave written informed consent in accordance with the Declaration of Helsinki. The protocol was approved by the Ethical Committee of the University of Lübeck (Az 11-198). Responsible members of the ethic committee: Prof. Dr. med. Dr. phil. Heiner Raspe and Prof. Dr. med. Frank Gieseler.

## Author Contributions

KŚ and RV contributed conception and design of the study. KW made data acquisition. KŚ and KW performed the statistical analysis. KŚ wrote the first draft of the manuscript. RV wrote sections of the manuscript. All authors contributed to manuscript revision, read and approved the submitted version.

## Conflict of Interest Statement

The authors declare that the research was conducted in the absence of any commercial or financial relationships that could be construed as a potential conflict of interest.

## Footnotes

1 www.easycap.de
